# The Identification of Host Proteins That Interact with Non-Structural Proteins-1α and -1β of Porcine Reproductive and Respiratory Syndrome Virus-1

**DOI:** 10.3390/v15122445

**Published:** 2023-12-16

**Authors:** Sofia Riccio, Kay Childs, Ben Jackson, Simon P. Graham, Julian Seago

**Affiliations:** 1 The Pirbright Institute, Ash Road, Pirbright, Woking GU24 0NF, UK; drsriccio@gmail.com (S.R.); kay.childs@pirbright.ac.uk (K.C.); ben.jackson@pirbright.ac.uk (B.J.); simon.graham@pirbright.ac.uk (S.P.G.); 2 Institute of Infection, Veterinary and Ecological Sciences, University of Liverpool, 146 Brownlow Hill, Liverpool L3 5RF, UK

**Keywords:** porcine reproductive and respiratory syndrome virus 1 (PRRSV-1), non-structural protein-1α (NSP1α), non-structural protein-1β (NSP1β), protein interactions, yeast-2-hybrid (y-2-h)

## Abstract

Porcine reproductive and respiratory syndrome viruses (PRRSV-1 and -2) are the causative agents of one of the most important infectious diseases affecting the global pig industry. Previous studies, largely focused on PRRSV-2, have shown that non-structural protein-1α (NSP1α) and NSP1β modulate host cell responses; however, the underlying molecular mechanisms remain to be fully elucidated. Therefore, we aimed to identify novel PRRSV-1 NSP1–host protein interactions to improve our knowledge of NSP1-mediated immunomodulation. NSP1α and NSP1β from a representative western European PRRSV-1 subtype 1 field strain (215-06) were used to screen a cDNA library generated from porcine alveolar macrophages (PAMs), the primary target cell of PRRSV, using the yeast-2-hybrid system. This identified 60 putative binding partners for NSP1α and 115 putative binding partners for NSP1β. Of those taken forward for further investigation, 3 interactions with NSP1α and 27 with NSP1β were confirmed. These proteins are involved in the immune response, ubiquitination, nuclear transport, or protein expression. Increasing the stringency of the system revealed NSP1α interacts more strongly with PIAS1 than PIAS2, whereas NSP1β interacts more weakly with TAB3 and CPSF4. Our study has increased our knowledge of the PRRSV-1 NSP1α and NSP1β interactomes, further investigation of which could provide detailed insight into PRRSV immunomodulation and aid vaccine development.

## 1. Introduction

Porcine reproductive and respiratory syndrome viruses (PRRSV) are responsible for one of the most important infectious diseases affecting the global pig industry [1]. PRRS is endemic in most major pig-producing countries where it is responsible for major economic losses [2,3]. PRRSV infects both pigs and wild boar, with the porcine alveolar macrophage (PAM) considered the primary target cell [4,5]. It causes mild to severe respiratory disease in new-born piglets and growing pigs and reproductive failure in sows, consequently impacting both growing and breeding sectors [6,7]. There are two species, PRRSV-1 and PRRSV-2, and both cause respiratory and reproductive disease, with PRRSV-2 generally more virulent than PRRSV-1 [8,9]. New strains are constantly evolving, including more virulent strains, allowing the virus to continue to evade the host immune response and re-infect pigs. In 2006, the Chinese swine industry was devastated following the emergence of a highly pathogenic PRRSV-2 strain, which killed pigs of all ages. Highly pathogenic PRRSV-1 strains have also been identified, such as the PRRSV-1 subtype 3 strains Lena [10] and SU1-Bel [5]. Both live attenuated and inactivated PRRSV vaccines are available and are widely used, but inactivated vaccines lack efficacy [11] and live attenuated vaccines have a poor safety profile due to their ability to revert to virulence and to recombine with wild-type viruses [12]. The huge economic costs demonstrate the global importance of PRRS and the need to increase our understanding of PRRSV to improve vaccines and other control strategies.

PRRSV is a member of the *Arteriviridae* family, order Nidovirales, and is an enveloped virus with a single stranded, positive sense, RNA genome [13,14]. The two PRRSV species, *Betaarterivirus suid 1* (PRRSV-1) and *Betaarterivirus suid 2* (PRRSV-2) [9,15], possess 55–70% homology at the nucleotide level [1]. The PRRSV genome is approximately 15 kb in length and is polycistronic with 11 open reading frames (ORFs), which code for at least 16 non-structural proteins (NSPs) and eight structural proteins [16]. For both PRRSV species, NSP2 is the most variable NSP with an amino acid identity of 24.4–28.0% due to its hypervariable region, which can accommodate large deletions [17]; between species, NSP9 is the most conserved NSP with an amino acid identity of 73.2% [16].

PRRSV NSPs are the first proteins to be produced during infection and are involved in viral replication and/or modulating the host immune responses [16]. NSP1α and NSP1β are translated from a single ORF as a polyprotein from which they self-cleave to produce discrete proteins [18]. NSP1α is required for sub-genomic RNA synthesis but not for genome replication [19], whereas NSP1β is required for the transactivation of programmed ribosomal frameshifting used to express NSP2TF [20]. Between them, NSP1α and NSP1β have been shown to modulate both innate and adaptive immune responses. NSP1α downregulates MHC class I surface expression [21], suppresses IFN-α production by causing CREB binding protein degradation [22], and inhibits both NF-κB activation and signalling [23,24]. NSP1β blocks host mRNA export [25], suppresses both TNF-α expression and inhibits its activity [24,26], and targets IFN-β in multiple ways [27,28]. However, the underlying molecular mechanisms remain to be fully elucidated, and most studies have focused on PRRSV-2 due to its prominence in North America and Asia. Therefore, this study aimed to identify novel PRRSV-1 NSP1–host protein interactions to improve our knowledge of NSP1-mediated immunomodulation.

## 2. Materials and Methods

### 2.1. Plasmids

A PAM complementary DNA (cDNA) library cloned into pACT2 (#638822, Takara Bio, Paris, France) was previously extracted from the respective lambda library and amplified. The lambda ACT2 library was originally constructed by Dr James Miskin [29].

cDNA sequences encoding NSP1α and NSP1β from a representative low virulence PRRSV-1 subtype 1 strain (UK field isolate 215-06, GenBank accession number (AC) OP047897) and the high virulence PRRSV-1 subtype 3 strain SU1-Bel (AC KP889243) [30] were amplified by PCR using the primers listed in Table 1 and cloned into pGBKT7 (Clontech, Takara Bio, Paris, France) between the *Nde*I and *Eco*RI restriction sites for expression in yeast as Gal4 DNA-binding domain fusion proteins.

cDNA sequences encoding full-length IFN-induced protein 35 (IFI35, AC XP_003358072.1), amino acids 27–275 of IFI35, and amino acids 1–179 of major vault protein (MVP, AC XP_020942083.1) were ordered and cloned into pMA-RQ (GeneArt, Thermo Fisher Scientific, Basingstoke, UK), with the restriction sites for *Nde*I and *Bam*HI added to the 5′ and 3′ ends, respectively, to facilitate cloning. Each open reading frame was excised from pMA-RQ and cloned into the *Nde*I and *Bam*HI sites of pGADT7 (Clontech). 

For the y-2-h screens, control plasmids were previously purchased as part of the Matchmaker GAL4 Two-Hybrid System 3 (Clontech) and amplified.

### 2.2. Primers

Table 1 details the primers used in PCR and sequencing (Merck, Gillingham, UK).

### 2.3. Plasmid Amplification

Two strains of *Escherichia coli* were used for plasmid amplification: *E. coli* JM109 (#L2005, Promega, Southampton, UK) and New England Biolabs (NEB) 10-beta Competent *E. coli* (High Efficiency) (#C3019H, NEB, Hitchin, UK). Bacteria were heat shock transformed and grown in sterile Luria lysogeny broth (LB) or plated on LB containing agar (LB agar) with or without the presence of antibiotic (ampicillin or kanamycin). Plasmids were extracted using the QIAprep Spin Miniprep Kit (#12123, Qiagen, Manchester, UK) or the QIAGEN Plasmid Midi Kit (#12143, Qiagen) according to the manufacturer’s protocol and eluted in nuclease/protease-free water (#W4502, Merck).

### 2.4. Polymerase Chain Reaction

PCR was used to amplify DNA for cloning, investigating the size and presence of plasmid inserts and to generate DNA samples for sequencing. For analysis, sequencing, and colony PCR, 1× ReadyMix™ Taq PCR Reaction Mix (#P4600, Merck) was used. For cloning, either GoTaq^®^ Long PCR Master Mix (#M4021, Promega) or Q5^®^ High-Fidelity DNA Polymerase (#M0494S, NEB) were used.

### 2.5. DNA Purification

PCR products, plasmids, and restriction fragments were purified or concentrated using the DNA Clean & Concentrator-5 kit (#D4004, ZYMO, Cambridge, UK) according to manufacturer’s protocol.

PCR products or restriction fragments were purified using agarose gel electrophoresis and the QIAquick Gel Extraction Kit (#28704, Qiagen) according to the manufacturer’s protocol.

### 2.6. Sanger Sequencing

Purified PCR samples and plasmids were sequenced using primers pGADT7 F and pGADT7 R (Table 1) and BigDye^®^ sequencing reagents (#4337454, Applied Biosystems, Waltham, MA, USA).

### 2.7. Yeast-2-Hybrid Assay

Yeast were transformed using the LiAc yeast transformation procedure according to the manufacturer’s instructions (PT3247-1, Clontech).

Tryptophan (Trp) or leucine (Leu)-deficient synthetic drop-out (SD) agar was used to check for successful transformation with pGBKT7 or pGADT7/pACT2 plasmids, respectively; SD agar additionally lacking adenine (Ade) and histidine (His) was used to check for activation of reporter genes. Additionally, where stated, the chromogenic substrate X-α-gal (#630463, Takara Bio) was added to SD agar plates (40 µg/mL) to detect the activity of the reporter α-galactosidase protein, as indicated by blue yeast colonies.

For the library scale transformations, yeast were sequentially transformed with the bait plasmid, either pGBKT7-215-06 NSP1α/β or pGBKT7-215-06 NSP1β, and then the pACT2-cDNA library. Due to self-cleavage, NSP1α/β expressed NSP1α fused to the transcription factor GAL4 binding domain (BD), as well as NSP1β.

Plasmids were extracted from positive colonies using the Easy Yeast Plasmid Isolation Kit (#630467, Takara Bio) following the manufacturer’s protocol and were eluted in nuclease/protease-free water (#W4502, Merck).

To identify putative interacting host proteins, the cDNA insert of each extracted prey plasmid was amplified via PCR using the pGADT7 F and pGADT7 R primers (Table 1). PCR samples were analysed by agarose gel electrophoresis to determine the number and size of amplified inserts and then sequenced. Sequences were translated and analysed using BLAST with the reference pig genome taxid: 9823 to identify host proteins.

To test the strength of interactions, positive colonies were streaked onto SD agar containing 3-amino-1,2,4-triazole (3-AT; #A8056, Merck) at concentrations ranging from 2.5 to 60 mM.

**Figure 1:**
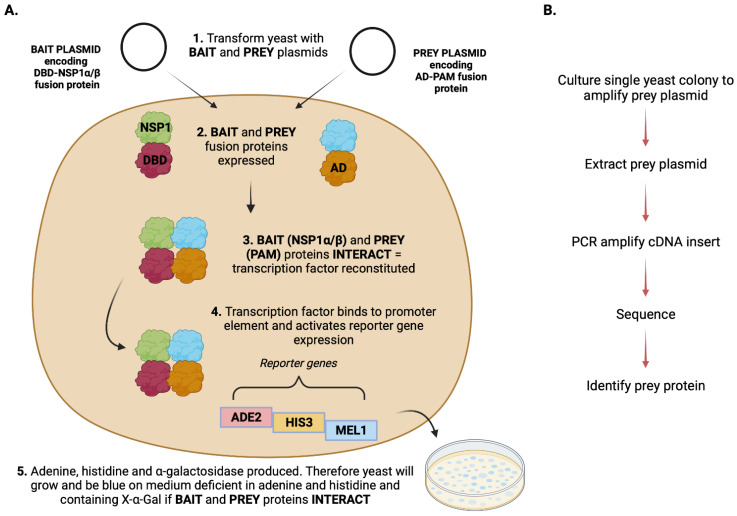
Overview of the y-2-h experimental approach to identify novel interactions. The y-2-h method with a pACT2-PAM-cDNA library was used to screen for putative interactions with PRRSV–1 NSP1α and NSP1β. (**A**) Yeast were transformed with both a bait plasmid, encoding the DBD–NSP1α/β fusion protein, and a prey plasmid, encoding the AD-PAM fusion protein. If the bait (NSP1α/β) and prey (PAM protein) interact, the transcription factor is reconstituted and binds to promoter elements. This activates reporter gene expression, resulting in adenine, histidine, and α-galactosidase production. Therefore, if bait and prey interact, yeast will grow and be blue on medium deficient in adenine and histidine and containing X-α-gal. (**B**) The subsequent workflow following a positive result. Individual yeast colonies were cultured to amplify their prey plasmids before extraction. Plasmid cDNA inserts were then PCR amplified and sequenced to identify PAM proteins. AD—transcription factor activation domain; DBD—transcription factor DNA-binding domain; PAM—porcine alveolar macrophage. Created with BioRender.com.

A schematic representation of the experimental approach to identify novel interactions is shown in Figure 1.

## 3. Results

### 3.1. Yeast-2-Hybrid Reveals Novel Interactions with PRRSV-1 NSP1α and NSP1β

To identify the host proteins that interact with PRRSV-1 NSP1α and NSP1β, we performed two high stringency y-2-h screens. Yeast were transformed with either pGBKT7-NSP1α/β or pGBKT7-NSP1β, and the pACT2-cDNA library, and plated onto a high-stringency medium (SD -Trp, -Leu, -Ade -His). Approximately 1.5 × 10^6^ yeast transformants were screened. Interactions with NSP1α were tested in the presence of NSP1β.

To identify the host proteins potentially interacting with NSP1α or NSP1β, the prey plasmids were extracted from the positive colonies and their cDNA inserts were sequenced. A maximum of approximately 200 putative interactions for each screen were selected to be investigated.

#### 3.1.1. Sixty Potential Binding Partners Were Identified in the PRRSV-1 NSP1α y-2-h Screen

**Figure 2:**
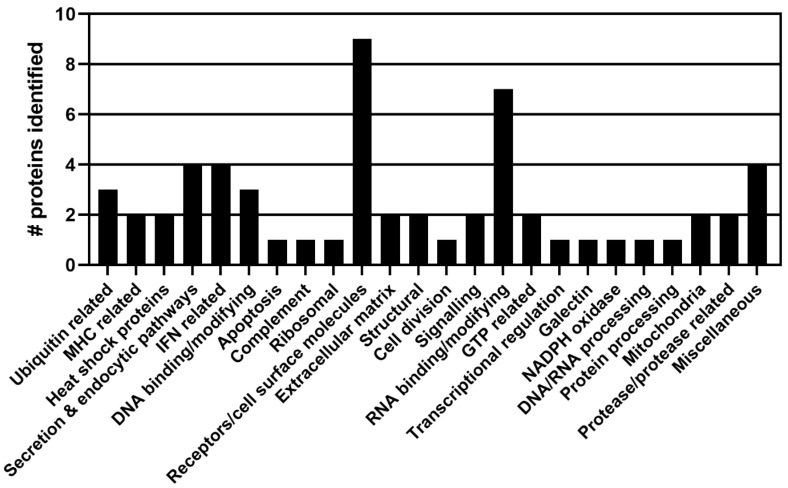
Proteins identified in the PRRSV-1 NSP1α y-2-h screen grouped by function. A y-2-h screen of NSP1α revealed 60 potentially interacting proteins, which were grouped broadly based on their known or putative cellular functions.

From the NSP1α screen, 88 sequences were obtained and analysed; these identified 60 potential binding partners for NSP1α. Table S1 shows the results of the NSP1α screen. The proteins were grouped based on their known or putative function, and Figure 2 shows an overview of the results and the numbers within each group.

#### 3.1.2. One Hundred and Fifteen Potential Binding Partners Were Identified in the PRRSV-1 NSP1β y-2-h Screen

**Figure 3:**
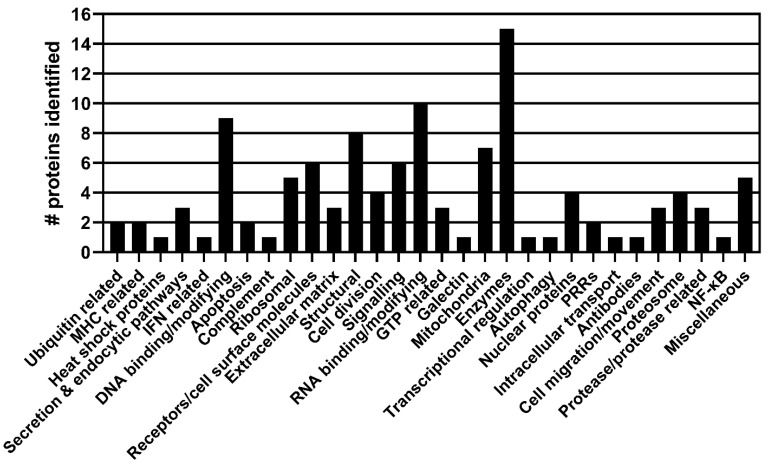
Proteins identified in the PRRSV-1 NSP1β y-2-h screen grouped by function. A y-2-h screen of NSP1β revealed 115 potentially interacting proteins, which were grouped broadly based on their known or putative cellular functions.

From the NSP1β screen, 233 sequences of cDNA inserts identified 115 potential binding partners for NSP1β. Table S2 shows the results of the NSP1β screen. The proteins were grouped based on their known or putative functions, and Figure 3 shows an overview of the results and the numbers within each group.

### 3.2. The Verification of Interactions between PRRSV-1 NSP1α and NSP1β and Selected Host Proteins

Many proteins identified in the NSP1α and NSP1β y-2-h screens are known to function in pathways targeted by PRRSV. Key pathways included nuclear transport, ubiquitination and the ubiquitin–proteasome pathway, the immune response, such as IFN signalling and the NF-κB pathway, and transcription and translation. NSP1β functions in both the cytoplasm and nucleus [25], but how it shuttles between the two is unknown. Proteins involved in transport across the nuclear membrane, such as nucleoporin GLE1 (GLE1) and nucleoprotein translocated promoter region (TPR), identified in the NSP1β screen (Table S2) could be involved in shuttling NSP1β. PRRSV alters the ubiquitome of the cell, although exactly how is unknown [31], and ubiquitin and the ubiquitin ligase MARCH7 were identified in the NSP1α screen (Table S1). Various proteasome subunits, including an immunoproteasome subunit, were identified in the NSP1β screen (Table S2). PRRSV targets multiple proteins for proteasomal degradation [21,32,33] and inhibits the immunoproteasome [34]. Based on these known associations, 13 proteins from the NSP1α screen and 31 proteins from the NSP1β screen that function in these pathways were selected for further analysis.

To confirm the interactions, yeast were co-transformed with either pGBKT7-NSP1α or pGBKT7-NSP1β, and an individual pACT2 plasmid isolated from the screens. Each pACT2 plasmid was also transformed into yeast with the empty vector pGBKT7 or pGBKT7 expressing p53 as a fusion protein as controls. As a positive control, yeast were transformed with pGBKT7-p53 and pGADT7 expressing the SV40 large T antigen (T).

#### 3.2.1. PRRSV-1 NSP1α Interacts with DNAJA3, PIAS1 and PIAS2

Of the thirteen proteins selected from the NSP1α screen for further analysis, three were confirmed to interact specifically with NSP1α in the absence of NSP1β (Table 2, Figures S1 and S3)**.**

Yeast co-transformed with the PIAS1 prey plasmid in combination with either the NSP1α or p53 bait plasmid grew and were equally blue. Yeast co-transformed with the PIAS2 prey plasmid in combination with either the NSP1α or p53 bait plasmid grew and were blue, although this was weaker with p53 in comparison to NSP1α. No growth was observed for yeast co-transformed with either the PIAS1 or PIAS2 prey plasmid and the empty pGBKT7 bait plasmid. Similarly, yeast co-transformed with DNAJA3 prey plasmid together with either the NSP1α or p53 bait plasmids grew and were blue, but there was minimal growth with the empty pGBKT7 bait plasmid. PIAS1, PIAS2, and DNAJA3 were tested with p53 as positive control, as all have previously been shown to interact with p53 [35,36,37]. These results confirm that PIAS1, PIAS2, and DNAJA3 interact with NSP1α.

The growth of control transformants was observed for yeast transformed with plasmids coding for either ubiquitin B, anaphase-promoting complex subunit 10, E3 ubiquitin-protein ligase MARCH7, p21 RAC1-activated kinase 2, cathepsin D, heat shock cognate 71 kDa protein, zinc finger MIZ-type containing 1, sialoadhesin precursor, and NHL repeat-containing 2 protein. Therefore, 9 out of 13 re-tested interactions were false positives.

#### 3.2.2. PRRSV-1 NSP1β Interacts with Twenty-Seven Host Proteins

Of the 31 proteins selected from the NSP1β screen for further analysis, 28 were confirmed to interact specifically with NSP1β (Table 3 and Figure S2).

On plates testing the interaction of NSP1β with either MHC Class II, cullin-9, TLR4, CPSF4, and CD163, yeast co-transformed with the respective PAM prey plasmid and NSP1β bait plasmid grew and were blue, but minimal growth was observed when co-transformed with the respective PAM prey plasmid and the empty pGBKT7 bait plasmid. Given how much stronger the yeast grew when expressing NSP1β, these interactions are genuine and the minimal growth with the empty pGBKT7 bait plasmid is background. On plates, including TAB3, STAT3, and beclin-1, yeast only grew and were blue when co-transformed with the PAM prey plasmid and the NSP1β bait plasmid; no growth was observed for the control transformants. Therefore, 27 out of the 31 proteins tested were confirmed to interact with NSP1β (Table 3).

Yeast co-transformed with prey plasmids coding for either V-Set and immunoglobulin domain-containing 4, integrin β2 precursor, proteasome maturation protein and high-temperature-requirement protein A2, and the control plasmids pGBKT7 or pGBKT7-53 all grew and were blue. Therefore, these interactions were false positives.

### 3.3. A Comparison of the Strength of Selected Confirmed Interactions

To investigate the strength of the interactions as well as reduce the background prey–protein transactivation of reporter genes, we performed y-2-h with 3-AT. 3-AT is a competitive inhibitor of the *HIS3* gene product, an enzyme required for the synthesis of histidine. The higher the 3-AT concentration, the stronger a prey–bait interaction must be to overcome it. The interactions were tested by streaking five colonies onto a high-stringency selection medium (-Trp, -Leu, -Ade, -His SD agar) containing increasing concentrations of 3-AT, ranging from 2.5 to 60 mM.

#### 3.3.1. PRRSV-1 NSP1α Interacts More Strongly with PIAS1 Than PIAS2 and DNAJA3

The strength of interactions between PRRSV-1 NSP1α and three host proteins was investigated using 3-AT: PIAS1, PIAS2, and DNAJA3. Table 4 summarizes the 3-AT results.

Yeast expressing NSP1α and PIAS1 grew strongly on all plates (Figure S3), but less strongly as the 3-AT concentration increased to 60 mM. The same growth pattern was seen in yeast expressing PIAS1 with p53, a known verified interaction [36]. There was no growth in yeast colonies transformed with the PIAS1 bait plasmid and empty pGBKT7 bait plasmid on any plates. Yeast co-transformed with PIAS2 prey plasmid and NSP1α bait plasmid grew on 10 mM 3-AT SD agar but not at higher 3-AT concentrations (Figure S4). As seen previously (Figure S1), there was also weak growth of yeast expressing PIAS2 and p53 on 10 mM 3-AT SD agar, albeit this interaction appeared comparatively weaker than the interaction between NSP1α and PIAS2. Yeast co-transformed with the DNAJA3 prey plasmid and any of the bait plasmids showed no growth on any 3-AT SD agar. As the growth of yeast transformed with plasmids expressing NSP1α and PIAS1 was observed on plates containing a higher 3-AT concentration than yeast transformed with plasmids expressing NSP1α and PIAS2, the interaction between NSP1α and PIAS1 is stronger than with PIAS2. As yeast transformed with plasmids expressing DNAJA3 and NSP1α did not grow at all in the presence of 3-AT, this interaction is the weakest of the three.

#### 3.3.2. PRRSV-1 NSP1β Interacts with TAB3 and CPSF4 on Agar Containing 2.5 mM 3-AT

The strength of the interactions between NSP1β and both TAB3 and CPSF4 was investigated using 3-AT (Table 4). Yeast were co-transformed with one of these prey plasmids in combination with either the empty pGBKT7, pGBKT7-53 or pGBKT7-NSP1β bait plasmid, and streaked onto SD agar deficient in Trp, Leu, Ade and His containing either 2.5 mM or 5 mM 3-AT (Figure S5).

Yeast transformed with the TAB3 prey plasmid and NSP1β bait plasmid grew on the 2.5 mM 3-AT SD agar plate, but not at the higher concentration of 5 mM 3-AT (Figure S5). Yeast expressing CPSF4 and NSP1β also grew in the presence of 2.5 mM but not 5 mM 3-AT (Figure S5). This confirmed the results previously observed (Figure S2) and suggests the interactions between NSP1β and either TAB3 and CPSF4 are equally as strong, but weaker than NSP1α interacting with PIAS1 (Figure S3) or PIAS2 (Figure S4).

#### 3.3.3. PRRSV-1 SU1-Bel NSP1α Interacts with PIAS1 Equally as Strongly as PRRSV-1 215-06 NSP1α

As well as transforming yeast with the PIAS1 prey plasmid and PRRSV-1 215-06 NSP1α bait plasmid, PIAS1 was also re-tested with NSP1α from the more virulent PRRSV-1 SU1-Bel strain. Yeast were plated on -Trp -Leu -Ade -His SD agar containing either 10, 20 or 60 mM 3-AT (Figure S3).

Yeast expressing SU1-Bel NSP1α and PIAS1 grew strongly on all plates (Figure S3), but less strongly as the 3-AT concentration increased to 60 mM; this is the same pattern observed between 215-06 NSP1α and PIAS1. Therefore, SU1-Bel NSP1α interacts with PIAS1, and this interaction has the same strength as the interaction between 215-06 NSP1α and PIAS1 at the 3-AT concentrations tested.

## 4. Discussion

PRRSV is endemic in most swine-producing countries, with the PRRS panzootic costing the swine industry millions every year [2,3]. This demonstrates the urgent need for safer and more effective PRRSV vaccines [38]. To achieve this, a deeper understanding of virus–host interactions involved in immunomodulation is required. PRRSV NSP1α and NSP1β have been shown to modulate the immune response to PRRSV in multiple ways, including inhibiting NF-κB activation, blocking type I IFN signalling pathways, and downregulating porcine MHC expression to reduce antigen presentation [23,25,39], as well as having crucial roles in viral replication [20]. However, the mechanistic details underlying immunomodulation are yet to be fully elucidated, and given the multifunctional nature of viral NSPs, it is likely they have other unidentified roles. NSP1α and NSP1β from the PRRSV-1 strain 215-06 were chosen for the study since most previous work has focused on PRRSV-2 strains, and 215-06 is a subtype 1 strain representative of the PRRSV-1 strains that predominate in western, central and southern Europe, North America and Asia.

Using the y-2-h method, 60 putative interactions with NSP1α (in the presence of NSP1β) and 115 interactions with NSP1β were identified. The identification of previously reported interactions, PIAS1 with NSP1α [40] and poly(rC)-binding protein 1 (PCBP1) with NSP1β [41], validates the results of these two screens. Three interactions from the NSP1α screen and twenty-seven interactions from the NSP1β screen were confirmed with additional experiments. The interacting host proteins are involved in either immune signalling, nuclear transport, ubiquitination, and transcription and translation, or have been shown to be targeted by PRRSV or other viruses. Further analysis found that NSP1α interacts very strongly with PIAS1 and strongly with PIAS2; in comparison, NSP1β was found to weakly interact with TAB3 and CPSF4. Only NSP1 from a single PRRSV-1 strain, except for testing the interaction between SU1-Bel NSP1α and PIAS1 (Figure S3), was tested. Therefore, y-2-h could be repeated using NSP1α and NSP1β from PRRSV-1 SU1-Bel, as well as from additional PRRSV-1 and -2 strains, to see if the reported interactions are conserved across subtypes and species, and if they correlate with virulence. Although the potential consequences of the confirmed interactions have not yet been investigated, we can postulate what they could be based on previously identified NSP1α and NSP1β functions, the pathways PRRSV targets, and knowledge from related viruses.

**Nuclear transport**: PRRSV replicates in the cytoplasm of infected cells [42], but NSP1α, NSP1β, and N protein also function in the nucleus [22,25,43]. Therefore, PRRSV needs to manipulate host nuclear transport machinery to shuttle these proteins between the two subcellular compartments. NSP1β translocation into the nucleus is dependent on karyopherin alpha 6 (KPNA6) [44]. Whilst no interaction between KPNA6 and NSP1β was observed in our or others’ screens [44], our y-2-h screens revealed the transcription factor STAT3 as an NSP1β-interacting protein. STAT3 is known to use KPNA6 to move into the nucleus [45]. Therefore, perhaps, STAT3 acts as a bridge to enable NSP1β to access the nucleus.

**Protein expression**: PRRSV downregulates and upregulates the expression of specific host genes during infection [46]. This could be to evade the immune response or increase levels of proteins required by PRRSV for successful replication. How PRRSV controls host gene expression has not been fully elucidated but could involve the targeting of host transcription factors (EPAS-1, MDFIC), transcription machinery (MED4, CPSF4), and translation regulators (GLE1), all of which were identified in the NSP1β y-2-h screen. Multiple ribosomal subunits, including 40S subunits, were also identified in both y-2-h screens. Given NSP1α and NSP1β are the first two viral proteins translated during infection [18], it is possible they bind to ribosomes and replication machinery to increase the production of other viral proteins and reduce host protein expression.

**Ubiquitination and the proteasome**: Ubiquitination is an intracellular protein modification that leads to either protein degradation via the 26S proteasome or regulates protein activity in many pathways [47,48,49]. PRRSV alters the ubiquitome of the cell, with 983 ubiquitination sites on 717 proteins shown to be altered [31], and requires a functioning ubiquitin-proteasome system for successful replication [50]. Interfering with ubiquitination in cells [31] would allow PRRSV to regulate the activation of multiple immune pathways and host protein degradation. Three ubiquitinated motifs in proteins have been shown to be targeted by PRRSV: K^ub^XXP, RXXXXLXK^ub^, and K^ub^Q, (where K = lysine; X = any amino acid; R = arginine; P = proline, Q = glutamine; L = Leucine) [31]. Interestingly, proteins identified in both y-2-h screens contained these motifs, notably DNAJA3 from the NSP1α screen and STAT3, TAB3, and proteasome subunits from the NSP1β screen. PSMA1 and STAT3 were previously shown to have altered ubiquitination during PRRSV infection [31]. The host ubiquitin ligases targeted by PRRSV have not been fully identified. Our y-2-h screens identified and confirmed kelch-like protein 20, a component of the (BTB-CUL3-RBX1) E3 ubiquitin–protein ligase complex [51], and the E3 ubiquitin ligase scaffold protein cullin-9 [52], as interacting with NSP1β. PRRSV upregulates the expression of PSMA1, a non-regulatory component of the proteasome, in infected cells [46], but suppresses the immunoproteasome, which processes peptides more efficiently for antigen presentation [53] by reducing the expression of the inducible subunits including PSMB8 [34]; both subunits were found to interact with NSP1β in this study. The selective control of proteasome subunit expression could allow PRRSV to increase the targeted degradation of host proteins whilst limiting antigen presentation. Whilst preparing this manuscript, Yi and colleagues reported that NSP1α interacts with PSMB4 via yeast-two-hybrid assay [54], suggesting that both NSP1α and NSP1β interact with PSMB4.

**Immune signalling pathways**: Our y-2-h screens identified multiple interactions with components of immune signalling pathways. PRRSV-1 NSP1β bound to cathepsin B and H that cleave TLR3 [55], which could be a strategy of PRRSV to prevent the formation of functional TLR3 signalling and avoid detection. Proteins involved in the type I IFN and NF-κB signalling pathways, PIAS1, PIAS2, STAT3, and TAB3, were identified in the y-2-h screens. NSP1α was confirmed to interact more strongly with PIAS1 than with PIAS2. PIAS1 and PIAS2 are SUMO ligases that regulate transcription factors involved in the immune response [56], including STATs and NF-κB [57,58]. TAB3, a regulator of both NF-κB activation and autophagy, interacts with NSP1β. TAB3 activates the NF-κB pathway by binding and activating TAK1 [59]. PRRSV has not been shown to target TAB3 previously but NSP1α [23,60], NSP4 [61] and NSP11 [62] do manipulate NF-κB signalling. Targeting these key antiviral pathways in multiple ways may allow PRRSV to replicate more efficiently. Autophagy is the lysosomal degradation pathway of cellular components and is important to PRRSV, as NSP2, NSP3, and NSP5 together induce incomplete autophagy in infected cells to increase viral replication [63,64]. We found that NSP1β binds to TAB3 and beclin-1. Beclin-1 regulates autophagy and is involved in inducing autophagosome formation [65], and TAB3 negatively regulates autophagy through binding beclin-1 [66]. It has been postulated that the autophagosome provides a replication site for PRRSV, as during infection, incomplete autophagy (no autophagosome and lysosome fusion) has been observed [63]. Inducing and regulating autophagy would provide PRRSV with a replication location shielded from pattern recognition receptors. PRRSV NSP1α and NSP4 target MHC class I for degradation [21,67] to reduce antigen presentation. The y-2-h screens identified that NSP1α interacted with the beta-2-microglobulin precursor, the part of the MHC class I complex whose expression is downregulated by PRRSV NSP4 [67], and confirmed that NSP1β interacts with porcine MHC class II. Reducing antigen presentation via MHC class I and II would impair cytotoxic and helper T cell responses, respectively.

## 5. Conclusions

The interactions identified and investigated in this study between host proteins and PRRSV-1 NSP1α or NSP1β have increased our knowledge of PRRSV-1 NSPs. This list, in combination with the future work characterizing the identified interactions, could help to reveal novel functions of the respective PRRSV proteins, as well as provide mechanistic detail to previously published immunomodulatory functions. This information would be crucial in rationally attenuating PRRSV for use in vaccines, and therefore in combating one of the economically most important infectious diseases affecting the global pig industry. 

## Figures and Tables

**Table 1:** DNA oligonucleotide primers used in PCR.

Primer Name	Sequence (5′ to 3′)
pGADT7 F	AACCACTGTCACCTGGTTG
pGADT7 R	ACAGTTGAAGTGAACTTGC
215-06 NSP1α F	GCGCATATGTCTGGGACGTTCTCC
215-06 NSP1α R	GCGGAATTCTTAATGAGCCTCTTC
215-06 NSP1β F	GCGCATATGTCCAGCGTGTACAGGTG
215-06 NSP1β R	GCGGAATTCTTAATACCACTTATG
SU1-Bel NSP1α F	GCGCATATGTCTGGGACGTTCTCC
SU1-Bel NSP1α R	GCGGAATTCTTAGTGGGCTTCCTC
SU1-Bel NSP1β F	GCGCATATGTCTGATGTGTATAAATG
SU1-Bel NSP1β R	GCGGAATTCTTAATACCACCTATG

Key: F—FORWARD primer; R—REVERSE primer.

**Table 2:** PRRSV–1 NSP1α interactions confirmed by y-2-h testing with additional control plasmids.

No.	AC ^#^	Protein *	Function	Interacting Amino Acid Region
1	XP_003354674.1	DNAJ homolog subfamily A member 3 (DNAJA3)	Molecular chaperone protein that binds and activates Hsp70 chaperone proteins to perform protein folding, degradation, and complex assembly. Also involved in maintaining mitochondrial DNA and membrane potential.	175–453
2	XP_020948317.1	E3 SUMO-protein ligase PIAS2 (PIAS2)	SUMO E3 ligase that regulates transcription factors, including STATs and NF-κB.	352–542
3	XP_003121797	E3 SUMO-protein ligase PIAS1 (PIAS1)	SUMO E3 ligase that regulates transcription factors, including STATs and NF-κB.	383–595

**^#^** GenBank accession number. * Protein abbreviation is listed in brackets.

**Table 3:** PRRSV-1 NSP1β interactions confirmed by y-2-h testing with additional control plasmids.

No.	AC ^#^	Protein *	Function	Interacting Amino Acid Region
1	NP_001231384.1	Proteasome subunit beta type-4 (PSMB4)	Non-catalytic subunit of the 20S proteasome complex.	1–264
2	AEX59168.1	MHC class II antigen, (MHC class II)	Antigen presentation	9–98
3	XP_020936488.1	TGF-beta-activated kinase 1 and MAP3K7-binding protein 3 (TAB3)	Regulates both the NF-κB pathway and autophagy.	414–701
4	BAM75557.1	IgG heavy chain precursor	The large polypeptide subunit of an antibody.	130–459
5	ACL97681.1	Toll-like receptor 4 (TLR4)	Pattern recognition receptor.	24–100
6	XP_020951815.1	Collectin-12	Soluble pattern recognition receptor that activates the alternative pathway of complement.	533–737
7	NP_001038045.1	Signal transducer and activator of transcription 3 (STAT3)	Transcription factor activated in response to cytokines and growth factors.	1–165
8	NP_001090927.1	Cathepsin B precursor	Lysosomal cysteine protease that functions in intracellular proteolysis.	44–327
9	XM_021062561.1	p21 (RAC1) activated kinase 1 (PAK1)	Protein kinase involved in intracellular signalling pathways downstream of integrins and receptor-type kinases.	76–179
10	XP_003122270.2	Nucleoporin GLE1 (GLE1)	RNA export mediator. Also involved in transcription termination and translation initiation and termination.	1–202
11	XP_003123013.1	Proteasome subunit alpha type-1 (PSMA1)	Non-catalytic subunit of the 20S proteasome complex.	74–263
12	XP_020946779.1	CD163	Receptor involved in clearance and endocytosis of haemoglobin/haptoglobin complexes by macrophages.	97–370
13	XP_013834181.1	Mediator of RNA polymerase II transcription subunit 4 (MED4)	Component of the mediator complex involved in regulating RNA polymerase II-dependent transcription.	1–104
14	AY792822.1	Cathepsin D protein	Lysosomal aspartic endo-protease that degrades proteins and activates protein precursors.	151–395
15	CAN13318.1	Proteasome subunit, beta type 8 (PSMB8)	Catalytic subunit of the immunoproteasome.	37–208
16	XP_001929303.2	Cullin-9	Hydrophobic protein that provides a scaffold for ubiquitin ligases.	296–458
17	NP_001090889.1	Endothelial PAS domain-containing protein 1 (EPAS-1)	Hypoxia-inducible transcription factor.	104–558
18	XP_020934777.1	MyoD family inhibitor domain-containing protein (MDFIC)	Acts as a transcriptional activator or repressor.	110–242
19	XP_013836386.1	Beclin-1	Regulates both autophagy and apoptosis.	5–448
20	ACB70169.1	Cathepsin H	Lysosomal cysteine proteinase important in the overall degradation of lysosomal proteins.	19–235
21	XP_020941778.1	Cleavage and polyadenylation specificity factor subunit 4 (CPSF4)	Component of the cleavage and polyadenylation specificity factor (CPSF) complex that functions in pre-mRNA 3′-end formation.	2–213
22	XP_013833352.2	Nucleoprotein TPR (TPR)	Component of the nuclear pore complex that regulates mRNA export via the NXF1:NXT1 pathway.	458–554
23	NP_001070681.1	Macrophage migration inhibitory factor (MIF)	Binds to CD74 on immune cells to trigger an acute immune response.	1–67
24	XP_005656773.1	Kelch-like protein 20	Component of the (BTB-CUL3-RBX1) E3 ubiquitin–protein ligase complex.	1–193
25	XP_020925047.1	Nuclear pore membrane glycoprotein 210 (NUP210)	Nucleoporin essential for nuclear pore assembly and fusion, nuclear pore spacing, as well as structural integrity.	655–721
26	NP_999472.1	10 kDa heat shock protein, mitochondrial (HSP10)	HSPs are chaperones with roles in folding/unfolding of proteins.	1–102
27	XP_020942083.1	Major vault protein (MVP)	Multi-subunit structures that may be involved in nucleo-cytoplasmic transport.	1–179

**^#^** GenBank accession number. * Protein abbreviation is listed in brackets.

**Table 4:** Growth of yeast transformants on agar containing increasing concentrations of 3-AT.

Protein Combination	No 3-AT	2.5 mM	5 mM	10 mM	20 mM	60 mM
NSP1α + PIAS1	+	+	+	+	+	+
PIAS1 + p53	+	+	+	+	+	+
NSP1α + PIAS2	+	+	+	+	-	-
PIAS2 + p53	+	+	+	+	-	-
NSP1α + DNAJA3	+	-	-	-	-	-
DNAJA3 + p53	+	-	-	-	-	-
NSP1β + TAB3	+	+	-	-	-	-
NSP1β + CPSF4	+	+	-	-	-	-

Key: + = growth; - = no growth.

## Data Availability

Data is contained within the article or Supplementary Material.

## Supplementary Materials

The following supporting information can be downloaded at: https://www.mdpi.com/article/10.3390/v15122445/s1. Figure S1: PRRSV-1 NSP1α interacts with DNAJA3 and PIAS2; Figure S2: Twenty-eight novel interactions between PRRSV-1 NSP1β and porcine proteins are genuine; Figure S3: PIAS1 interacts with NSP1α from PRRSV-1 215-06 and SU1-Bel strains on agar containing 60 mM 3-AT; Figure S4: PIAS2 interacts with PRRSV-1 NSP1α on agar containing 10 mM 3-AT; Figure S5: TAB3 and CPSF4 interact with PRRSV-1 NSP1β on agar containing 2.5 mM 3-AT; Table S1. Proteins identified in the NSP1α yeast-2-hybrid screen; Table S2. Proteins identified in the NSP1β yeast-2-hybrid screen.
