# Supplementary material for: The Identification of Host Proteins That Interact with Non-Structural Proteins-1α and -1β of Porcine Reproductive and Respiratory Syndrome Virus-1
Source: Viruses. 2023 Dec 16;15(12):2445. doi: 10.3390/v15122445 (PMC10747794; doi:10.3390/v15122445)
Supplement: Supplementary file 1 [file viruses-15-02445-s001.zip › viruses-2745551-supplementary/Supplementary Figures SR.pdf]

The Identification of host proteins that interact with non-structural proteins -1α and -1β of porcine reproductive and respiratory syndrome virus-1

Sofia Riccio, Kay Childs, Ben Jackson, Simon P. Graham, Julian Seago

Supplementary Figures

A

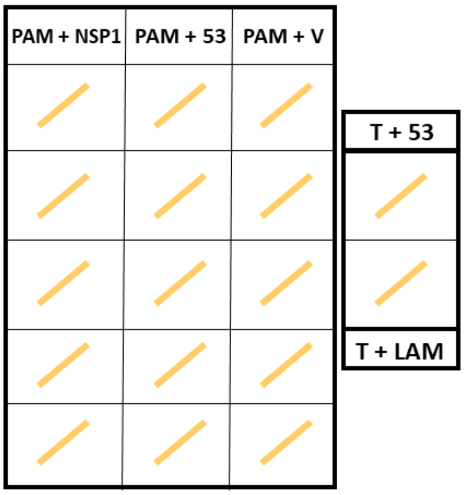

B

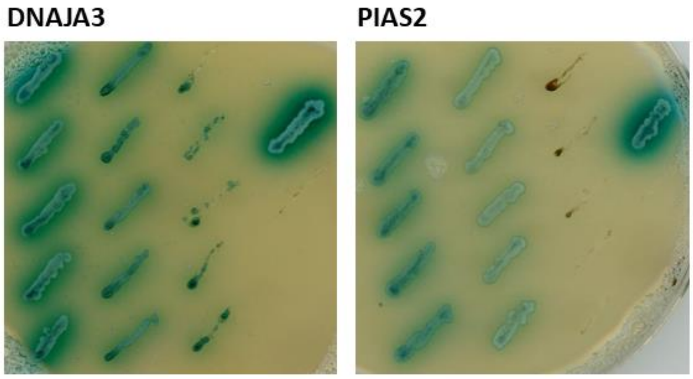

Figure S1: PRRSV-1 NSP1α interacts with DNAJA3 and PIAS2. Plate layout (**A**) for each yeast plate in (**B**). Yeast that had been co-transformed with combinations of the respective prey plasmid (termed "PAM protein" in (**A**)) and the bait plasmids NSP1α, pGBKT7-53 and pGBKT7 (termed "NSP1", "53" and "V" respectively in (**A**)) were plated on high stringency selection medium (SD agar -Trp, -Ade, -Leu and -His) containing X-α-Gal. Each streak corresponds to a different colony. The appearance of blue growth by yeast transformants expressing NSP1α and the respective PAM protein ("PAM protein + NSP1" in (**A**)) suggested an interaction. However, the interaction was considered a possible false positive if blue growth was also observed for the control transformant ("PAM protein + V" in (**A**)). Yeast co-transformed with pGADT7-T and pGBKT7-53 together served as the positive control, and pGADT7-T and pGBKT7-LAM as the negative control.

A

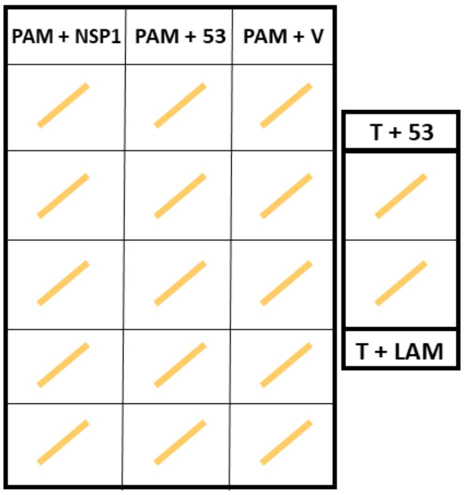
PAM + NSP1
PAM + 53
PAM + V
T + 53
T + LAM

B

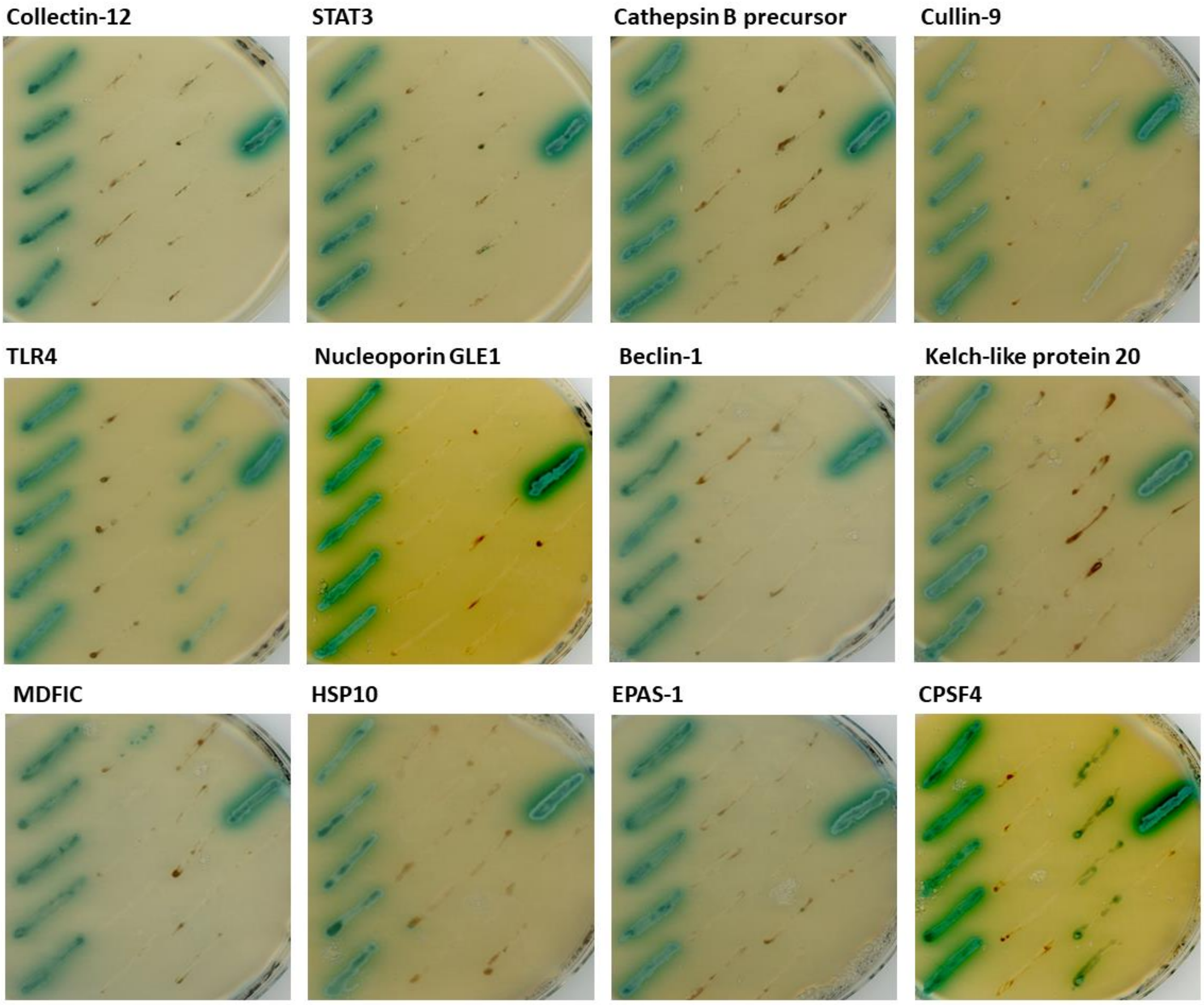
Collectin-12
STAT3
Cathepsin B precursor
Cullin-9
TLR4
Nucleoporin GLE1
Beclin-1
Kelch-like protein 20
MDFIC
HSP10
EPAS-1
CPSF4

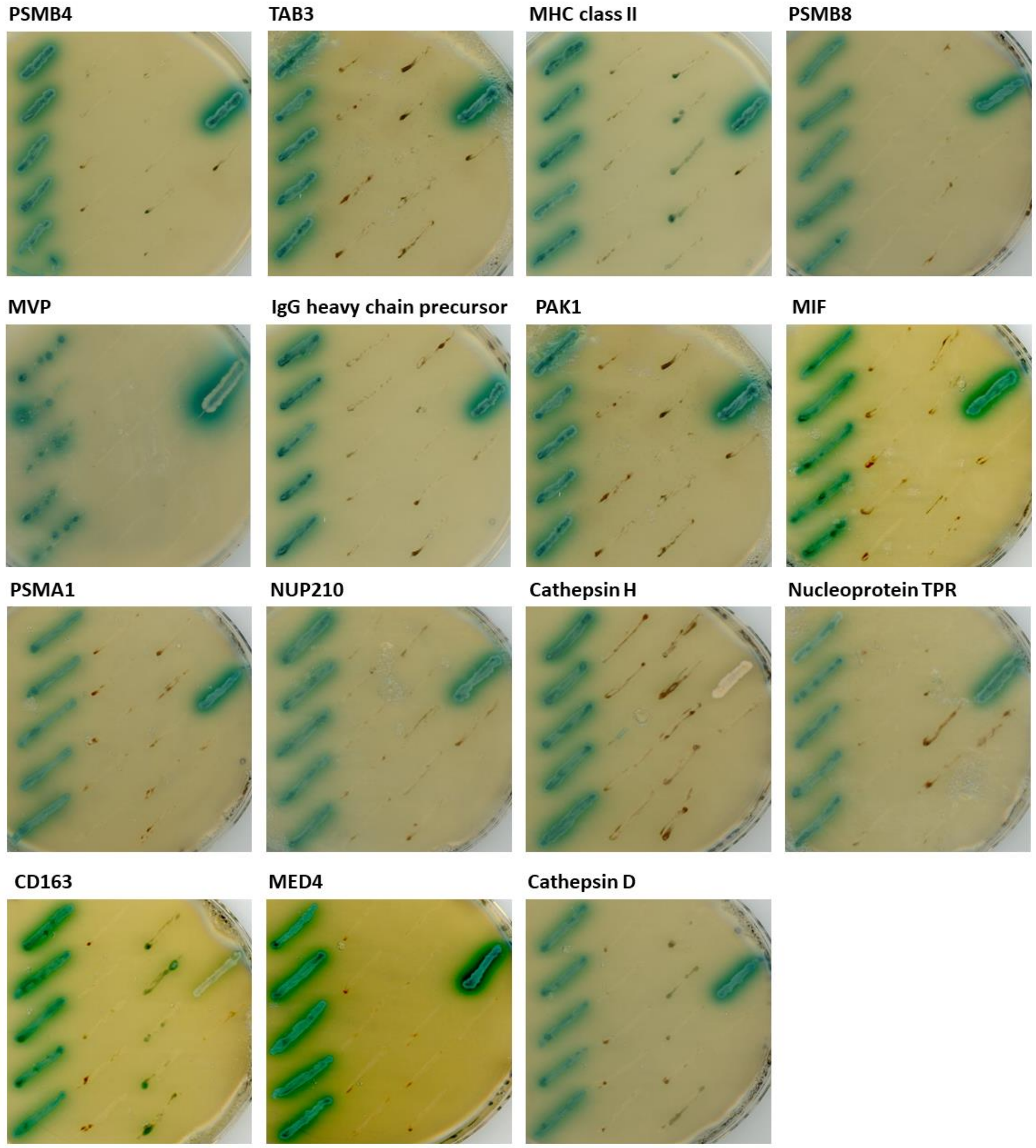

Figure S2: Twenty-eight novel interactions between PRRSV-1 NSP1β and porcine proteins are genuine. Plate layout (**A**) for each yeast plate in (**B**). Yeast that had been co-transformed with combinations of the respective prey plasmid (termed "PAM protein" in (**A**)) and the bait plasmids NSP1β, pGBKT7-53 and pGBKT7 (termed "NSP1", "53" and "V" respectively in (**A**)) were plated on high stringency selection medium (SD agar -Trp, -Ade, -Leu and -His) containing X-α-Gal. Each streak corresponds to a different colony. The appearance of blue growth by yeast transformants expressing NSP1β and the respective PAM protein ("PAM protein + NSP1" in (**A**)) suggested an interaction. However, the interaction was considered a possible false positive if blue growth was also observed for the control transformants ("PAM protein + 53" and "PAM protein + V" in (**A**)). Yeast co-transformed with pGADT7-T and pGBKT7-53 together served as the positive control, and pGADT7-T and pGBKT7-LAM as the negative control.

A

		PAM + V	PAM + 53	PAM + SU1-Bel NSP1	PAM + 215-06 NSP1
T + 53					
T + LAM					

B

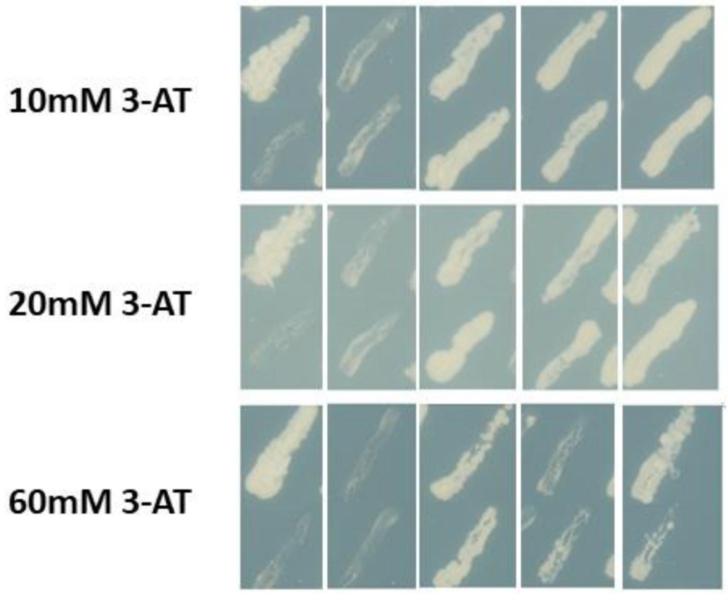

Figure S3: PIAS1 interacts with NSP1α from PRRSV-1 215-06 and SU1-Bel strains on agar containing 60 mM 3-AT. Plate layout (**A**) for each yeast plate in (**B**). Yeast that had been co-transformed with combinations of the PIAS1 prey plasmid (termed "PAM protein" in (**A**)) and the bait plasmids NSP1α, pGBKT7-53 and pGBKT7 (termed "NSP1", "53" and "V" respectively in (**A**)) were plated on high stringency selection medium (SD agar -Trp, -Ade, -Leu and -His) containing either 10 mM, 20 mM or 60 mM 3-AT (**B**). Each streak corresponds to a different colony. The appearance of growth by yeast transformants expressing NSP1α and the respective PAM protein ("PAM protein + NSP1" in (A)) suggested an interaction; the higher the 3-AT concentration of the SD agar the yeast grew on, the stronger the interaction. Yeast co-transformed with pGADT7-T and pGBKT7-53 together served as the positive control, and pGADT7-T and pGBKT7-LAM as the negative control.

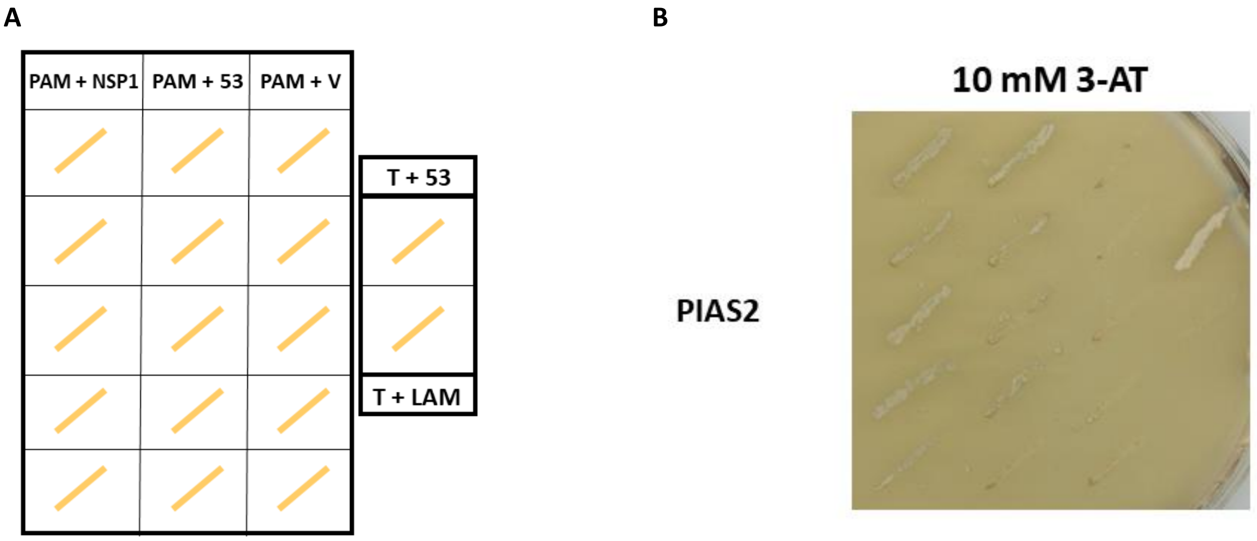

Figure S4: PIAS2 interacts with PRRSV-1 NSP1α on agar containing 10 mM 3-AT. Plate layout (**A**) for yeast plate in (**B**). Yeast that had been co-transformed with combinations of the respective prey plasmid (termed "PAM protein" in (**A**)) and the bait plasmids NSP1α, pGBKT7-53 and pGBKT7 (termed "NSP1", "53" and "V" respectively in (**A**)) were streaked on high stringency selection medium (SD agar -Trp, -Ade, -Leu and -His) containing either 10 mM, 20 mM or 60 mM 3-AT. Each streak corresponds to a different colony. The appearance of growth by yeast transformants expressing NSP1α and the respective PAM protein ("PAM protein + NSP1" in (**A**)) suggested an interaction; the higher the 3-AT concentration of the SD agar the yeast grew on, the stronger the interaction. Yeast co-transformed with pGADT7-T and pGBKT7-53 together served as the positive control, and pGADT7-T and pGBKT7-LAM as the negative control.

A **B**

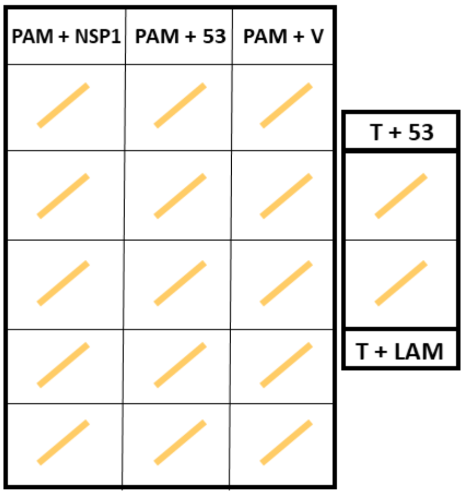

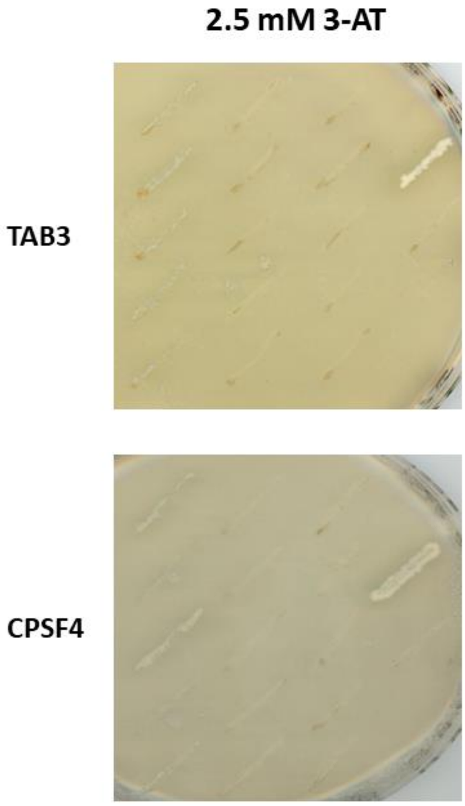

Figure S5: TAB3 and CPSF4 interact with PRRSV-1 NSP1β on agar containing 2.5 mM 3-AT. Plate layout (**A**) for each yeast plate in (**B**). Yeast that had been co-transformed with combinations of the respective prey plasmid (termed "PAM protein" in (**A**)) and the bait plasmids NSP1β, pGBKT7-53 and pGBKT7 (termed "NSP1", "53" and "V" respectively in (**A**)) were plated on high stringency selection medium (SD agar -Trp, -Ade, -Leu and -His) containing either 2.5 mM or 5 mM 3-AT. Each streak corresponds to a different colony. The appearance of growth by yeast transformants expressing NSP1β and the respective PAM protein ("PAM protein + NSP1" in (**A**)) suggested an interaction; the higher the 3-AT concentration of the SD agar the yeast grew on, the stronger the interaction. Yeast co-transformed with pGADT7-T and pGBKT7-53 together served as the positive control, and pGADT7-T and pGBKT7-LAM as the negative control.
